# THBS1 as a candidate biomarker and fibrotic mediator in radiation-induced liver injury: insights from TMT-labeled quantitative proteomics

**DOI:** 10.3389/fphar.2025.1659870

**Published:** 2025-11-21

**Authors:** Zixi Wang, Tong Wu, Haixu Wang, Yawen Deng, Jing Liu, Tingting Wang, Xue Ren, Ying Sun, Haibo Zhang, Defu Yang, Feng Shang, Ying Xu, Dongyang Lv, Ying Yan

**Affiliations:** 1 Department of Radiation Oncology, General Hospital of Northern Theater Command, Shenyang, China; 2 Graduate School of Dalian Medical University-General Hospital of Northern Theater Command, Dalian, China; 3 Key Laboratory on Signaling Regulation and Targeting Therapy of Liver Cancer, Ministry of Education, Eastern Hepatobiliary Surgery Hospital/National Center for Liver Cancer, Naval Medical University, Shanghai, China; 4 Department of Abdominal Radiation Oncology Ward I, Cancer Hospital of Dalian University of Technology, Shenyang, Liaoning, China; 5 Beifang Hospital of China Medical University, Shenyang, China

**Keywords:** proteomics, TMT, JAK2/STAT3, THBS1, biomarkers

## Abstract

**Objective:**

Radiation-induced liver injury (RILI) is one of the most dreaded complications in radiotherapy for hepatocellular carcinoma (HCC), causing serious impact on the course of treatment and the survival quality of patients. This study was conducted to screen effective biomarkers for the diagnosis and disease course monitoring of RILI.

**Methods:**

This study established a rat model of RILI, with the assessment of liver injury by hematoxylin-eosin (HE) staining. High-throughput screening of RILI and normal liver tissue samples was performed using TMT quantitative proteomics technology, followed by the analysis of differentially expressed proteins (DEPs) using GO and KEGG. Weighted gene co-expression network analysis (WGCNA) and protein-protein interaction (PPI) network analysis were further employed to identify THBS1 as a key protein of RILI. We knocked down THBS1 in rat (BRL, BRL-3A) and human (THLE-2) hepatocytes using siRNA and applied Ruxolitinib to inhibit the JAK2/STAT3 pathway, further clarifying the role of THBS1 in this signaling process. Validation was performed by protein-protein docking and Western blot. The concentration of THBS1 in plasma was determined using enzyme linked immunosorbent assay (ELISA), while the consistency of plasma and tissue expression was analyzed by Pearson’s correlation analysis.

**Results:**

Proteomic analysis identified 176 DEPs, of which 106 were upregulated, with THBS1 identified as a key protein highly expressed in RILI. THBS1 could activate the PDGFA/PDGFR signaling pathway, which in turn leads to the activation of the JAK2/STAT3 pathway, resulting in the deposition of COL5A and COL6A. Silencing THBS1 with siRNA in BRL, BRL-3A, and THLE-2 cells significantly reversed the activation of the JAK2/STAT3 signaling pathway and the overexpression of collagens in the cellular models. In addition, plasma ELISA revealed that the concentration of THBS1 in plasma increased with increasing radiation dose and degree of RILI, which was consistent with the expression level in the liver tissue.

**Conclusion:**

This study provides new insights into the pathogenesis of RILI, and identifies THBS1 as a potential biomarker for RILI diagnosis and monitoring.

## Graphical Abstract

**Figure:**
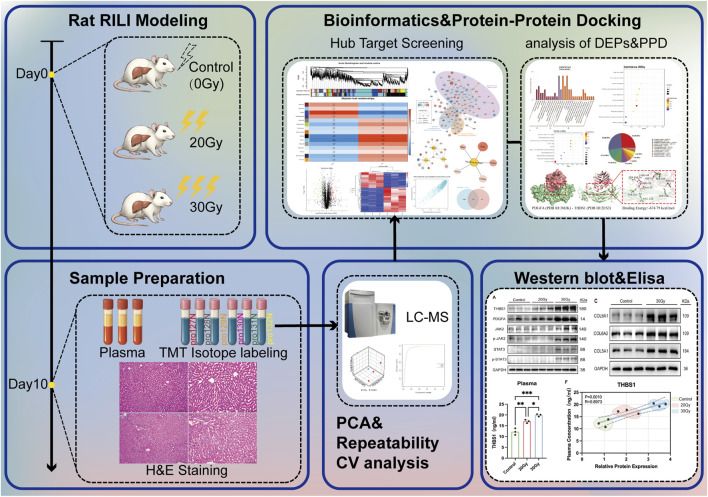

## 1 Introduction

Radiotherapy is one of the three major therapeutic options for human tumors. Currently, stereotactic radiation therapy has become a promising modality for the treatment of hepatocellular carcinoma (HCC), which is frequently adopted to manage and control tumor metastases for patients with intermediate and advanced HCC that cannot undergo surgery (Sharma et al., 2025; Dudzinski et al., 2025). Nevertheless, almost all radiotherapy strategies are not immune to the emergence of radiotoxic side effects. Radiation-induced liver disease (RILI), proposed for the first time by Ingold, is one of the most serious complications of radiotherapy (Raza and Sood, 2014). Patients may usually present with main manifestations such as abdominal discomfort, hepatomegaly and jaundice-free ascites (Lia et al., 2011). Since both HCC cells and normal hepatocytes are radiosensitive, the liver is highly susceptible to radiation exposure and RILI during radiotherapy for HCC. Radiotherapy is more likely to cause RILI when applied for patients with cirrhosis and hepatic insufficiency concurrently. Moreover, the rest of tumors treated with upper abdominal radiotherapy may also develop severe RILI after radiotherapy due to the proximity of the liver (Munoz-Schuffenegger et al., 2017). Furthermore, RILI can occur as early as 2 weeks (hepatocyte edema, degeneration, and necrosis-dominated) and as late as 7 months (liver fibrosis and cirrhosis-dominated) after radiotherapy (Saha et al., 2024). The prevailing mechanistic theories of RILI include the direct and indirect effects of ionizing radiation (IR), including the direct effect of radiation triggering DNA breakage, and the indirect effect of reactive oxygen species produced by radiation in combination with water on various key proteins involved in life activities (Gawish et al., 2024; Valeriani et al., 2023). Programmed apoptotic cell death is generally initiated by the combined effect of both (Obrador et al., 2022; Song et al., 2025). However, there is still a poor understanding of the mechanisms that ultimately lead to RILI-attributable fibrosis. The effective dose of radiotherapy against most solid malignant tumors is 50–70Gy (Zhou et al., 2023). However, about 5%–10% risk of RILI already exists when the liver tissue is exposed to 30–35Gy (Kim et al., 2017a). The risk of developing RILI climbs dramatically as the radiation dose rises, and it reaches at least 50% when the liver is exposed to 60Gy of radiation (Kim et al., 2017b). In addition, severe RILI is prone to rapid complications of liver failure (Li et al., 2021), RILI may produce significant negative impact on the treatment and survival of HCC patients receiving radiotherapy. Therefore, in order to guide the early diagnosis and disease monitoring of RILI, it is critical to study the mechanism of RILI and screen reliable biomarkers.

RILI is a complex multifactorial pathological process. Exposure to IR may activate the expression of platelet-derived growth factor A (PDGFA) in hepatic epithelial cytosol, inducing the formation of dimers that exert potent inflammatory chemotaxis (Ouyang et al., 2018). Activated Kupffer cells (KC) can boost the release of pro-inflammatory cytokines (e.g., TGF-β, IL-6 and IL-1β) (Zhou et al., 2012), which may further increase collagen synthesis by activating the Notch and JAK/STAT signaling pathways (Shi et al., 2024). Simultaneously, damaged hepatic sinusoidal endothelial cells can initiate the coagulation cascade, accompanied by activated TGF-β1-Smad2/3 signaling pathway after radiation exposure, eventually driving collagen deposition and liver tissue remodeling (Saha et al., 2024). Existing evidence has documented the role of inactivating the JAK/STAT pathway in significantly attenuating radiation-induced inflammatory responses, thereby preventing liver fibrosis (Gawish et al., 2024; Galal et al., 2018).

Thrombospondin-1 (THBS1) is a secreted glycoprotein found mainly in blood and extracellular matrix (ECM), occupying an important position in several biological processes (BP) of RILI, including promoting hepatic inflammatory response, mediating acute and chronic liver failure, and fibrin deposition (Gutierrez and Gutierrez, 2021; Hassan et al., 2022). THBS1 was first extracted and characterized as a platelet membrane-sensitive protein in 1971, and is secreted extracellularly upon platelet activation, thus involving in platelet degranulation and regulating coagulation factor activity (Baenziger et al., 1971). Prior research based on single-cell RNA sequencing and experimental validation has revealed the pivotal role of THBS1 in collagen deposition (Xiong et al., 2024).In addition, multiple molecules have been identified to regulate collagen synthesis and deposition in the ECM by targeting and regulating THBS1 expression, and, in turn, collagen deposition (Yang et al., 2024; Liu B. et al., 2024; Cao et al., 2024).

Proteins are executors of all BP, which are directly involved in various phenotypic changes. There may be corresponding alteration in the complex network of proteins in the context of RILI, resulting in a new equilibrium. Therefore, it underlines the significance of exploring the molecular mechanism of RILI occurrence at the protein level by employing proteomics. Tandem mass tagging (TMT)-based proteomics is a high-throughput protein quantification technique, the core principle of which is to label peptides in different samples with different chemical tags to give them different mass markers in mass spectrometry analysis, thus enabling comparison of proteins from multiple samples in the same analysis (Li et al., 2025).

Through the establishment of a rat RILI model, the present study was performed to identify and elucidate the important role of THBS1 in regulating ECM collagen deposition in RILI by applying applied TMT-based proteomics.

## 2 Materials and methods

### 2.1 Animals and rat RILI modeling

All experiments in our study were approved by the Ethics Committee for Animal Experiments of the General Hospital of the Northern Theater of Operations of the Chinese People’s Liberation Army (2023M734296). A total of 33 healthy male rats (weighing 160–230 g, Liaoning Changsheng Biologicals) were used and randomly divided into the control (3 rats), 20 Gy (15 rats), and 30 Gy (15 rats) groups. Prior to subsequent processing, all rats were anesthetized using inhaled isoflurane after fasting for 2 h. Rats in the experimental groups were irradiated with a single 20 Gy and a single 30 Gy using the Infinity linear gas pedal of Medtronic, and a source-skin distance of 100 cm, respectively. During irradiation, rats were well aligned with the localization outline (Supplementary Figures S1A–E). Rats in the control group were anesthetized only without irradiation. One hour after irradiation, all rats resumed normal feeding and drinking. Three rats in each group were executed 10 days later to harvest the livers (Supplementary Figures S1F, G), half of which were immediately immersed in saline, cut into small pieces, placed in a freezing tube and stored on ice to prevent protein denaturation, and ultimately stored in a −80 °C refrigerator. The other half of the liver tissue was fixed by 4% neutral paraformaldehyde and then paraffin tissue embedded to produce 5 μm tissue sections. To verify the successful establishment of the rat RILI model, liver sections were subjected to hematoxylin-eosin (HE) staining for pathological examination. The proportion of inflammatory cell infiltration area and the proportion of fibrosis (blue staining) area were analyzed using ImageJ software.

### 2.2 Protein sample preparation

A portion of rat liver tissue preserved at −80 was ground into powder, added to PASP protein lysis solution (100 mM NH4HCO3, 8M Urea, pH 8), and lysed by ultrasonication in an ice water bath for 5 min. Then, the obtained samples were centrifuged at 4 °C and 12,000 g for 15 min to collect the supernatant. After that, another 1 h of reaction was performed at 56 °C after the addition of 10 mM DTT, followed by another reaction for 1 h at room temperature in the dark when IAM was added. Subsequently, 4x volume of pre-cooled acetone were added to precipitate at −20 °C for 2 h. The generated precipitate was collected by centrifugation, followed by repeated precipitation with acetone. After air-drying, the proteins were solubilized using a proteolytic solution for quantification of proteins using a Bradford kit.

### 2.3 TMT labeling

After the lysis of proteins, trypsin and 100 mM TEAB buffer were added for subsequent 4 h of digestion at 37 °C. Another overnight digestion was performed with the addition of trypsin and CaCl_2_. The pH was adjusted with formic acid to less than 3. The next steps were centrifugation after mixing, de-salting using a C18 column, as well as washing and elution with 70% acetonitrile. The samples were lyophilized and dissolved in TEAB, followed by reaction at room temperature for 2 h after the supplementation of TMT labeling reagent. The reaction was terminated with ammonia, with the reaction mixture desalted and lyophilized finally.

### 2.4 Fraction separation and liquid chromatography-mass spectrometry (LC-MS)

LC-MS was performed with the preparation of mobile phase A (2% acetonitrile, and 98% water) and mobile phase B (98% acetonitrile, and 2% water). The lyophilized powder was dissolved with liquid A, centrifuged and passed through a Waters BEHC18 column (4.6 × 250 mm, 5 μm) for gradient separation at a column temperature of 45 °C. With the collection of one tube per minute, it was subjected to lyophilization and dissolution with formic acid. Mass spectrometry was performed using a TMT tag with a mass spectral range of 350–1,500 m/z, primary resolution of 60,000, secondary resolution of 45,000, and fragmentation collision energy of 32% (Supplementary Figures S2A, B).

### 2.5 Bioinformatics analysis

The identified proteins were analyzed for Gene Ontology (GO), InterPro (IPR), Cluster of Orthologous Groups of Proteins (COG) as well as Kyoto Encyclopedia of Genes and Genomes (KEGG) functions and pathways using InterProScan software. DEPs were analyzed for volcano map, GO, IPR, KEGG and subcellular localization. Furthermore, to identify key modular genes, clustering analysis was performed by WGCNA package in R software (version 1.73). Protein-protein interaction (PPI) networks were constructed using the STRING database (https://string-db.org) and Cytoscape 3.9, associated with functional annotation by the Reactome database. PPD was performed using the HDOCK 1.1 software, and the number of docking samples was set to 100,000. Finally, the top-10 results with the best binding energy were exported, visualized and analyzed using PyMOL 2.6.

### 2.6 Hematoxylin and eosin (H&E) staining

Paraffin-embedded sections of rat liver tissues were deparaffinized in xylene for 10 min (×2), passed stepwise through 100%, 95%, 80%, and 70% ethanol for 5 min of hydration each time, and finally immersed in distilled water for 5 min. Then, the processed sections were immersed in hematoxylin staining solution for 5 min, rinsed under running water for 5 min, and differentiated with 1% hydrochloric acid in alcohol for 10–20 s. After rinsing under running water for another 5 min, these sections were finally blued with ammonia or lithium carbonate for 30 s. After that, sections were stained in 1% eosin solution for 1–2 min, rinsed under running water, and then dehydrated in 70%, 80%, 95%, and 100% ethanol for 2 min, respectively. Finally, the sections were transparent in xylene for 10 min (×2) and sealed with neutral resin.

### 2.7 Cell culture

Rat hepatocyte lines (BRL and BRL-3A) were obtained from iCell (Shanghai, China) and cultured in Dulbecco’s Modified Eagle’s Medium (DMEM, Gibco, China) supplemented with 10% fetal bovine serum (FBS, Gibco, United States) and 1% penicillin/streptomycin. The human hepatocyte line (THLE-2) was purchased from ATCC (Manassas, VA, United States; catalog numbers CRL-2706 and CRL-11233) and maintained in BEGM SingleQuots medium (iCell, Shanghai, China) containing 10% FBS.

### 2.8 siRNA transfection and chemicals

For gene silencing, small interfering RNA (siRNA) oligonucleotides specifically targeting THBS1 were obtained from GenePharma (Suzhou, China). Cells were seeded at an appropriate density and transfected with siRNA using Lipofectamine™ 2000 (Life Technologies, Thermo Fisher Scientific, MA, United States) following the manufacturer’s instructions. The final siRNA concentration was 37.5 nM. After 48 h of transfection, cells were exposed to 10 Gy of irradiation, with a non-targeting siRNA used as the negative control. Transfection efficiency was verified by Wb. For *in vitro* inhibition experiments, Ruxolitinib (S1378, Selleck Chemicals, United States) was dissolved in DMSO and diluted to a final concentration of 5 μM. Cells were treated with Ruxolitinib for 48 h, followed by 10 Gy irradiation, and protein samples were collected 48 h post-irradiation for analysis.

### 2.9 Western blot (WB)

Total protein concentration was determined by BCA protein quantification kit (Coolaber, China) as instructed. For protein separation, the SDS-PAGE was performed on 10% polyacrylamide gel (with 12% separation gel). Then, 20 μg of quantitative protein sample was taken and mixed with loading buffer, boiled at 95 °C for 5 min, and stored at 4 °C for use. The gel was electrophoresed at 80 V for separation until the dye front reached the bottom of the gel. The proteins were transferred to PVDF or nitrocellulose membranes at 300 mA for 1.5 h and cooled at 4 °C. Membranes were sealed with 5% skim milk powder for 2 h of incubation at room temperature. Then, these membranes were incubated with specific primary antibody overnight at 4 °C and secondary antibody for 2 h at room temperature. After 3 times of washing (7 min each) using TBST, membranes were treated with ECL luminescent substrate (Thermo Scientific Pierce ECL), and signals were captured using a chemiluminescent imaging system (Bio-Rad ChemiDoc or GE Amersham Imager). Quantitative analysis of the protein bands was performed by ImageJ to calculate the relative expression of the target proteins, with normalization to the internal reference GAPDH. Specific information of the antibodies (i.e., company, stock number, and dilution ratio) used for WB are listed in the Supplementary Material.

### 2.10 Enzyme linked immunosorbent assay (ELISA)

ELISA was performed (THBS1; Animaluni, Shanghai; 0.156–10 ng/mL) to determine the concentration of THBS1 in plasma. The plasma samples of 0 Gy, 20 Gy, and 30 Gy groups were diluted 5-fold for the assay, and the absorbance was measured at 450 nm by a microplate spectrophotometer (Supplementary Figures S2E).

### 2.11 Immunohistochemical staining (IHC)

Paraffin-embedded rat liver sections were dewaxed in xylene, rehydrated through graded ethanol, and treated with 3% hydrogen peroxide to block endogenous peroxidase. After blocking with 5% BSA, sections were incubated overnight at 4 °C with the THBS1 primary antibody (1:2000). A HRP-conjugated secondary antibody was then applied, followed by DAB visualization and hematoxylin counterstaining. Slides were mounted with neutral resin and examined under a Carl Zeiss optical microscope.

### 2.12 Data analysis

Data were searched and obtained using ProteomeDiscoverer 2.4 with the rattus_norvegicus_uniprot database, with a precursor mass tolerance of 10 ppm and a fragment mass tolerance of 0.02 Da, as well as modifications including cysteine alkylation, methionine oxidation, and TMT tagging. Results were filtered using a False-Detection-Rate (FDR) to remove peptides and proteins with FDR >1%. Meanwhile, DEPs were analyzed using T-test (P < 0.05, Log2FC > 0.55 or < -0.55). GraphPad Prism 10 (San Diego, CA, United States) was employed for data analysis and result visualization. Normally distributed measurements were expressed as mean ± standard deviation (SD). Inter-group comparisons were made using independent samples t-tests. A value of P < 0.05 was considered statistically significant (*P < 0.05, **P < 0.01, and ***P < 0.001).

## 3 Result

### 3.1 Establishment of rat RILI model and pathologic evidence

After radiation exposure, rats in the 30Gy group showed significantly decreased body weight loss compared to that of rats in the 20Gy group (average weight loss: 5.20 g vs. 1.98 g). All three rats in the control group showed a gradual increase in body weight. All mice survived from the beginning of irradiation to the 10th day after irradiation. Rats in the 30Gy group had dull fur from the third day onwards, a large asymmetric hair loss in the irradiated field on day 4, and localized erythema on the skin, accompanied by pain reactions such as licking, scratching of the irradiation field. Rats in the 20Gy group showed hair thinning and localized hair loss from day 7 onwards, with smooth and slightly erythematous skin, none significant pain reaction of licking and scratching. Furthermore, 5 rats in the 30Gy group showed obvious diarrhea (Bristol 6 type) on day 4, and the remaining 10 rats showed obvious diarrhea (Bristol 6 type) on day 5, with feces adhering to the anus in all rats. In addition, rats in the 30Gy group had obviously decreased food intake compared with that of the 20Gy group; and only two rats in the 20Gy group showed mild diarrhea on day 5 (Bristol type 5).

**FIGURE 1:**
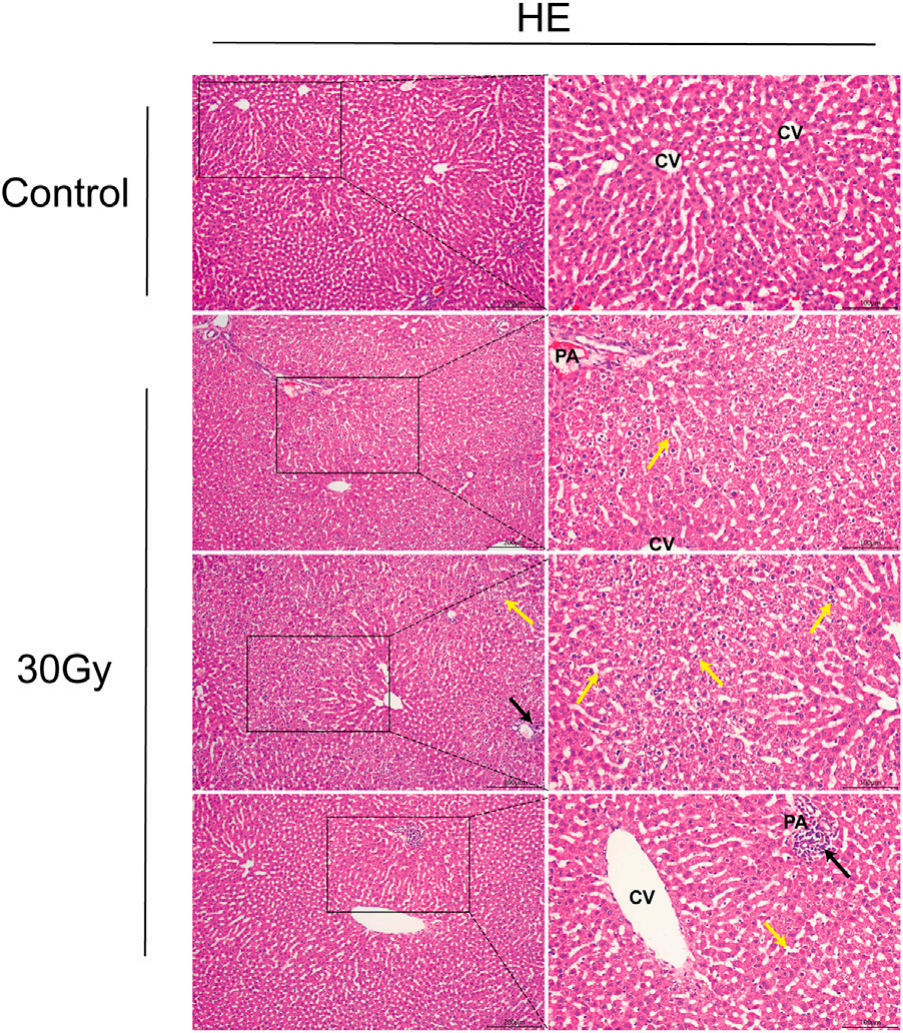
HE stains of liver tissue control group vs. 30Gy group. Cell swelling (Yellow arrows) and inflammatory infiltration (Black arrows) in hepatocytes. PA: Portal areas; CV: Central vein.

Based on the results of HE staining, on the 10th days after radiation exposure, rats were exhibited pathologic changes of liver specimens under light microscope. One of the three rats in the 20 Gy group showed hydropic degeneration of hepatocytes, and all of the three rats in the 30 Gy group showed varying degrees of hydropic degeneration of hepatocytes and inflammatory infiltration. The control group showed no obvious pathological changes in the liver sample. It indicated successful establishment of an early stage RILI model in rat (Figure 1). Given that all three samples in the 30Gy group showed different degrees of pathological changes, the rat liver samples from this group were selected for subsequent proteomic analysis and screening for DEPs.

### 3.2 Inter-group differences in variability and repeatability levels based on principal component analysis (PCA) and coefficient of variation (CV) cumulative curves

**FIGURE 2:**
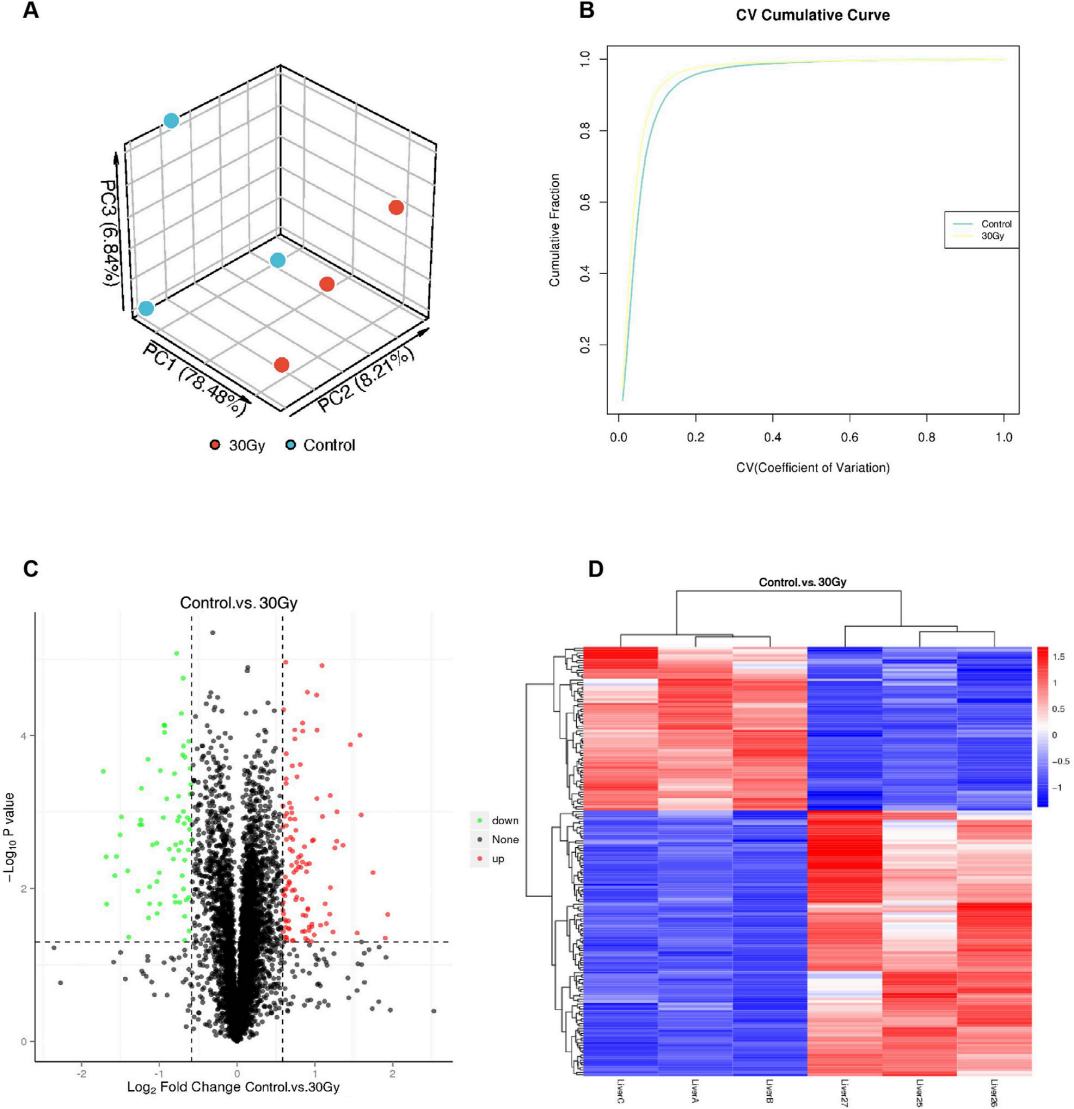
Proteomic profiling and quality assessment of differential protein expression between RILI and control liver tissues. **(A)** PCA: Principal Component Analysis; **(B)** Repeatability CV analysis; **(C,D)** Volcano plot and heat map of DEPs between RILI tissue and normal lung tissue.

PCA showed significant separation in the gene expression profiles of the 30Gy group and the control group in three-dimensional space, especially in the PC1 direction. Therefore, there might be obvious differences between the two groups in gene expression or proteomic features. The relative intra-group clustering of the three samples indicated relative spatial consistency in terms of the main characteristics of samples within group, suggesting low variability and good reproducibility in intra-group samples (Figure 2A). The CV cumulative curve can facilitate the assessment of the difference in inter-group variability, where the X-axis with lower CV indicates a higher concentration of the sample value, and higher CVs indicate greater inter-sample variation. Meanwhile, the Y-axis indicates the cumulative fraction, i.e., the proportion of samples with different CV values and below. As the CV increases, the cumulative fraction elevates and eventually approaches 1, indicating 100% of the sample data. As shown in Figure 2B, the steeper CV cumulative curve for the RILI and the control groups revealed less variability in the sample at 100%, i.e., better repeatability of the overall samples.

### 3.3 Screening of key gene THBS1 by protein differential analysis and WGCNA

**FIGURE 3:**
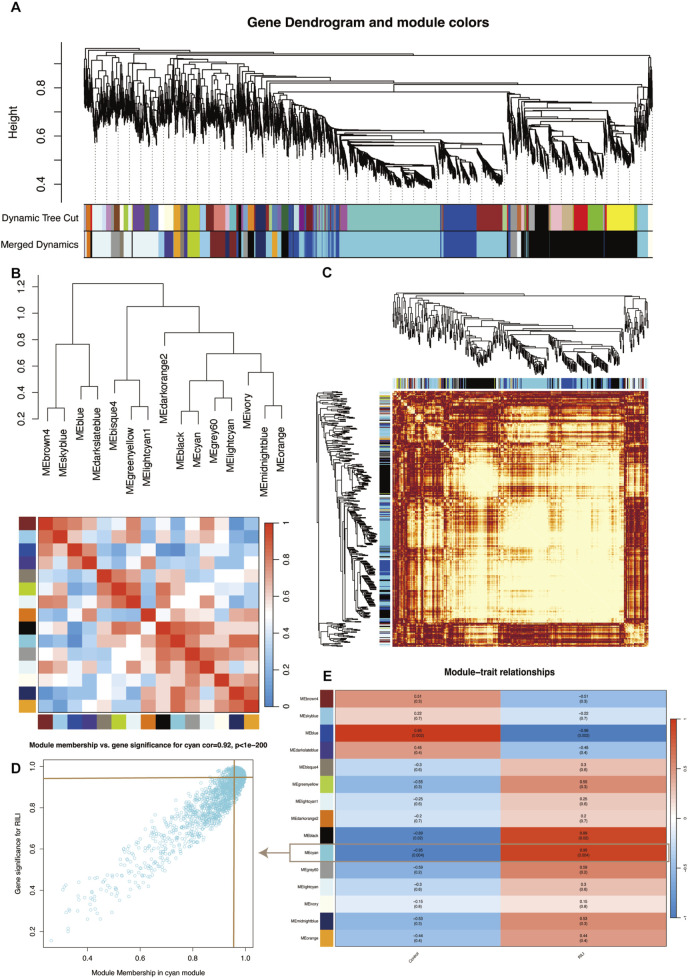
WGCNA identifying RILI-associated gene modules and their correlation with phenotypic traits. **(A)** Gene clustering dendrogram with module partitioning; **(B)** Similarity clustering between module eigenvalues with heatmaps; **(C)** Topological overlap matrix (TOM) heatmap; **(D)** Plot of gene significance within a module in relation to module membership. The point labeled by the crossing line is THBS1; **(E)** Plot of gene significance within a module in relation to module membership.

Weighted Gene Co-expression Network Analysis (WGCNA) was performed to identify gene modules closely associated with the radiation-induced liver injury (RILI) phenotype. As shown in Figures 3A–C, genes were clustered based on their expression profiles, with those exhibiting similar patterns grouped into the same module. Hierarchical clustering identified 15 distinct gene modules, among which the Cyan module showed the strongest correlation with RILI (R = 0.95, Figures 3D,E). Liver tissues from the modeled and control rats were taken for proteomics, resulting in 176 DEPs, of which 106 were upregulated and 70 were downregulated. These DEPs were visualized using volcano and heat maps (Figures 2C,D). By Venn diagram analysis, THBS1 gene was enriched in both WGCNA key modules and DEGs list, with both MM and GS of >0.85, suggesting its potentially significant role in the development of RILI with high biological significance (Supplementary Figures S2D). The key DEPs intersected with the WGCNA (Cyan) are shown in Table 1, mainly including THBS1, STAT3, COL6A2, etc.

**TABLE 1:** Key genes at the intersection of DEPs and WGCNA (Cyan).

Gene	Description	P Value	Log2FC	MM	GS
THBS1	Thrombospondin 1	0.0437	0.6333	0.9752	0.9417
STAT3	PhospholipaseA2,membrane associated	0.0003	0.7704	0.9688	0.9886
COL6A2	Collagen type VI alpha-2 chain	0.0081	0.6676	0.8708	0.8694
COL6A1	Collagen type VI alpha 1 chain	0.0043	0.5810	0.8935	0.9498
COL5A1	Collagen type V alpha-1 chain	0.0322	0.5520	0.8784	0.9285

### 3.4 Functional enrichment highlights ECM remodeling and structural protein activation

**FIGURE 4:**
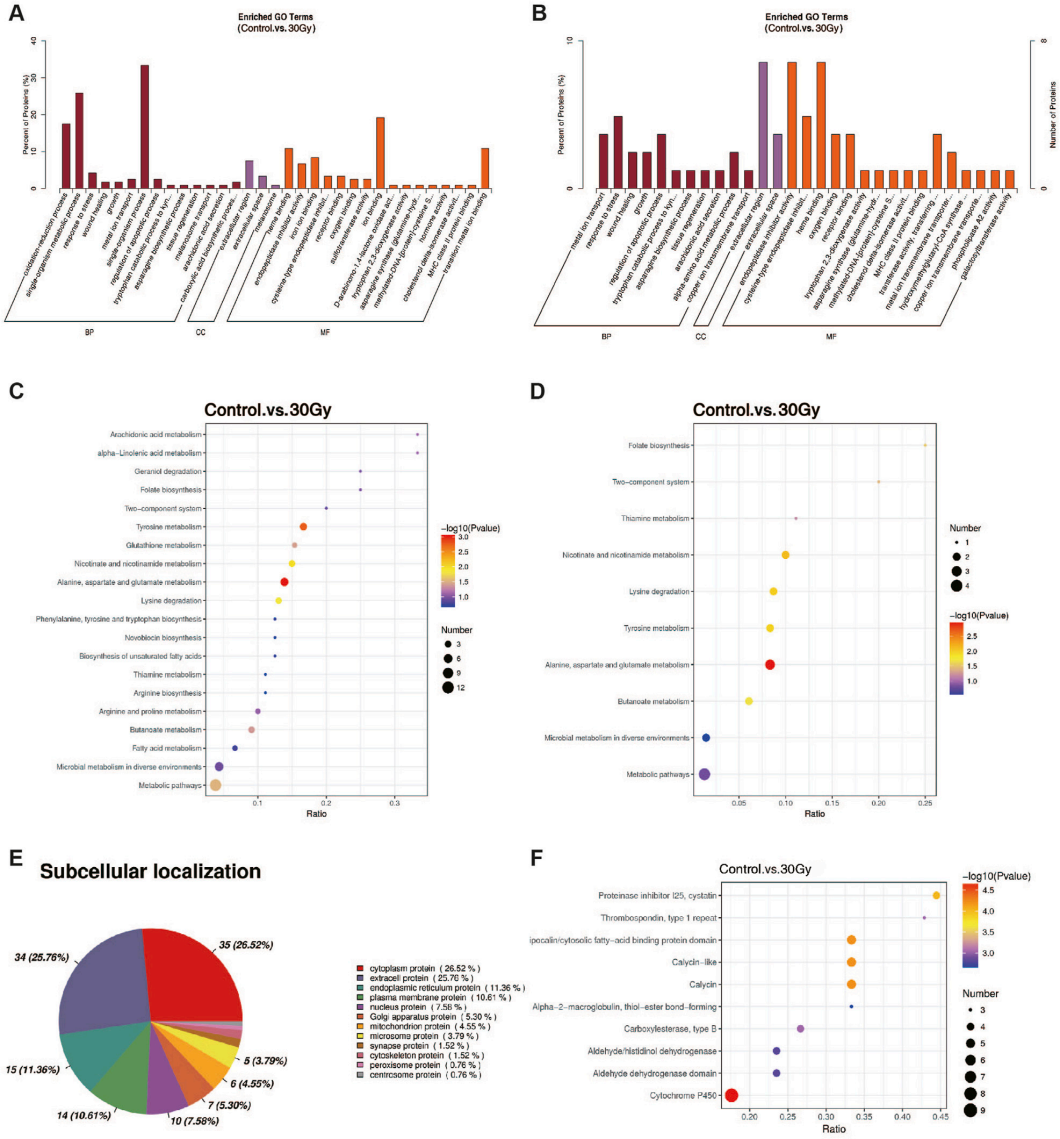
Functional enrichment and subcellular localization analyses of DEPs in RILI. **(A)** GO enrichment analysis of DEPs; **(B)** GO enrichment analysis of upregulated DEPs; **(C)** KEGG enrichment analysis of DEPs; **(D)** KEGG enrichment analysis of upregulated DEPs; **(E)** Analysis of the subcellular localization of DEPs: More than 1/4 of the DEPs are distributed in the cytoplasm or extracellular matrix; **(F)** Structural domain enrichment analysis of DEPs: The biological roles of DEPs, as highly conserved functional units, can be revealed by structural domain enrichment analysis at both structural and functional levels. Enriched functional modules or protein family features can be effectively identified, in turn indicating the activation or inhibition of potential signaling pathways.

To elucidate the biological functions of the DEPs following radiation exposure, multiple enrichment analyses were performed, including GO, KEGG, domain annotation, and subcellular localization. Collectively, the results indicated that radiation-induced changes in the liver proteome were closely associated with ECM remodeling, stress response, and metabolic adaptation.In the GO enrichment analysis (Figures 4A,B), biological process (BP) terms were predominantly enriched in wound healing, response to stress, and extracellular matrix organization, suggesting enhanced liver tissue remodeling and repair activities after irradiation. Cellular component (CC) terms were highly concentrated in the extracellular region and plasma membrane, aligning with the molecular function (MF) enrichment in endopeptidase inhibitor activity, receptor binding, and metal ion binding. These results indicate that radiation primarily activated extracellular and secreted proteins involved in ECM structure and intercellular signaling. Such enrichment patterns are consistent with the biological behavior of structural matrix proteins like collagens (COL family) and matricellular regulators such as THBS1, both of which contribute to ECM integrity and remodeling. KEGG pathway analysis (Figures 4C,D) further demonstrated that DEPs were mainly involved in ECM–receptor interaction, focal adhesion, and amino acid metabolism (particularly alanine, aspartate, glutamate, and tyrosine pathways). These metabolic pathways are essential for collagen synthesis and oxidative stress regulation, implying that radiation-induced metabolic reprogramming may support ECM reconstruction and fibrotic progression.

Subcellular localization analysis (Figure 4E) revealed that over one-quarter of DEPs were distributed in the extracellular space and plasma membrane. This finding corroborates the idea that major proteomic alterations occurred in the extracellular microenvironment. Structural domain enrichment analysis (Figure 4F) revealed significant enrichment of the thrombospondin type I repeat domain, a THBS1 hallmark, as well as of protease inhibitor I25. These results emphasize the roles of anti-proteolytic and matrix-binding motifs in ECM stabilization and tissue remodeling. These results imply that RILI triggers a coordinated response centered on ECM reorganization. Upregulation of ECM-associated domains and localization patterns implies that key structural proteins, including THBS1 and collagens, play central roles in mediating matrix remodeling and stress adaptation during RILI repair.

### 3.5 THBS1 serves as a central hub linking ECM remodeling and PDGF-JAK-STAT signaling

**FIGURE 5:**
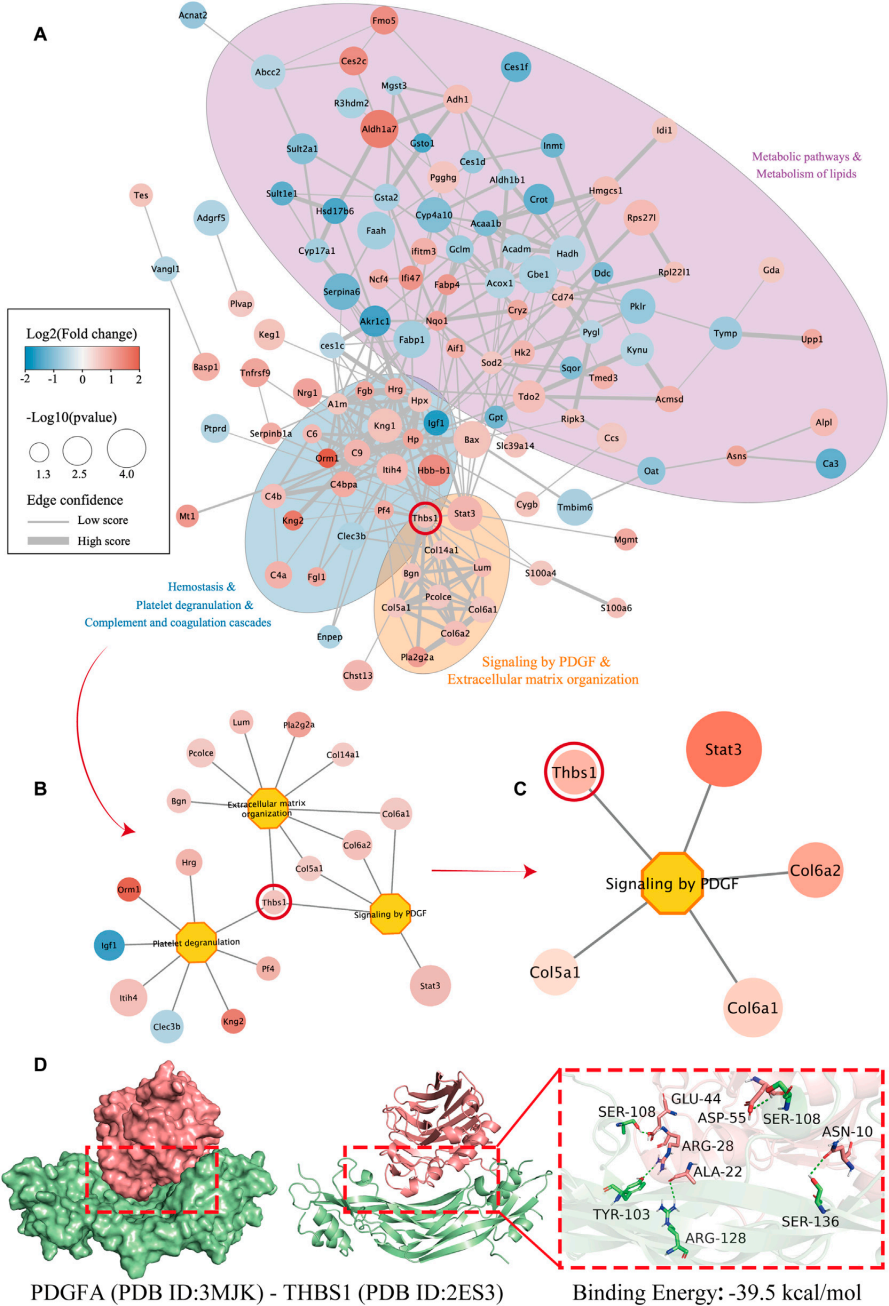
PPI network and molecular docking reveal THBS1 as a central regulator linking PDGF signaling and extracellular matrix remodeling in RILI. **(A)** PPI analysis and result visualization via CytoScape. DEPs are visualized using a PPI-based analysis, where node size indicates statistical significance (-log10 p value), color displays changes in expression (red: upregulation, and blue: downregulation), and edge thickness reflects the level of confidence in the interaction. Multiple functional clusters can be identified in the network, including lipid metabolism and metabolic pathways (purple region), hemostasis and platelet degranulation (blue region), as well as PDGF signaling and extracellular matrix organization (orange region). THBS1 is positioned as a core node connecting multiple important functional clusters, suggesting its key role in multi-pathway regulation; **(B)** Further extraction of proteins directly interacting with THBS1 and their related biological processes to form a functional sub-network centered on “extracellular matrix remodeling” and “PDGF signaling pathway”. THBS1 is enriched in the extracellular matrix regulatory pathway together with Col6a1 and Col6a2, suggesting its important function in tissue remodeling and inflammatory repair; **(C)** Identification of the closely related signaling molecules and their downstream targets by focusing on the key pathway “Signaling by PDGF”, such as Stat3 and Col6a2, to further validate the possible mechanism of THBS1 mediating tissue fibrosis and inflammation through regulating PDGF signaling; **(D)** The protein-protein docking results of PDGFA (green, PDB ID: 3MJK) with THBS1 (pink, PDB ID: 2ES3). There are several interactions between key residues in the binding interface, including ARG-28, ARG-128, SER-108, TYR-103, etc. The binding energy is as high as −39.5 kcal/mol, suggesting that the two factors may form a stable complex, providing potential theoretical support for further verification of their molecular interactions.

PPI analysis revealed that radiation-induced DEPs were mainly enriched in metabolic pathways, platelet degranulation, ECM organization, and PDGF signaling (Figure 5A). THBS1 occupied a central hub position within this network, bridging the ECM structural module (COL6A1, COL6A2, and COL5A1) and the signaling module (the PDGF–STAT3 axis) (Figures 5B,C). This topological configuration suggests that THBS1 acts as a key mediator that links extracellular matrix remodeling to the activation of intracellular profibrotic signaling.

### 3.6 Identification of potential binding sites for THBS1 and PDGFA

Molecular docking analysis further supported the physical and functional relevance of this interaction network. The THBS1–PDGFA complex exhibited a stable binding conformation (binding energy = −39.5 kcal/mol), involving key residues such as SER108, ARG28, and ASP55 (Figure 5D). This high-affinity binding implies that THBS1 can facilitate PDGFA engagement and amplify downstream signaling, reinforcing its regulatory role in ECM remodeling and profibrotic signaling cascades.

### 3.7 THBS1 is markedly upregulated in rat liver tissue following radiation-induced liver injury

**FIGURE 6:**
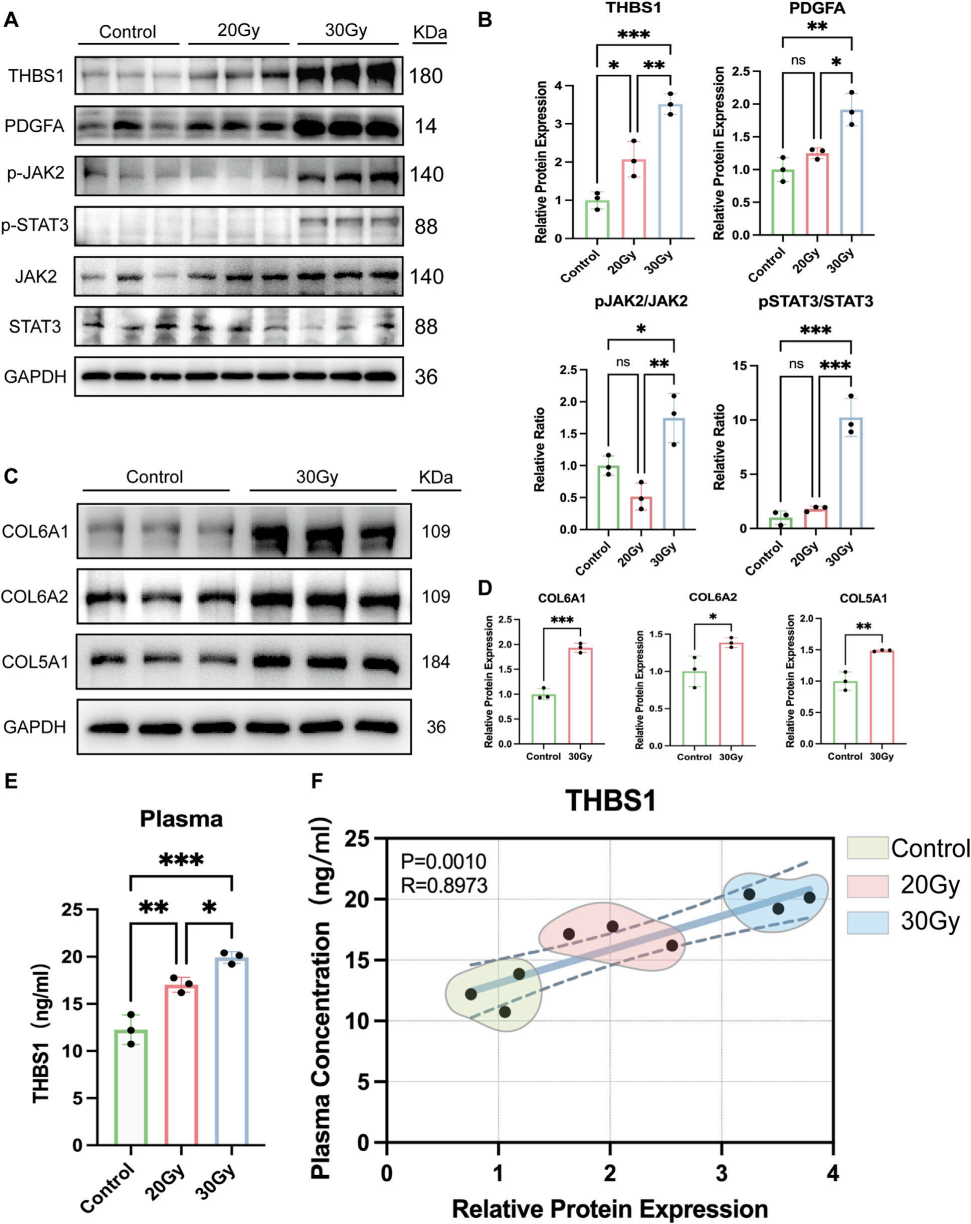
Validation of THBS1 upregulation and activation of the PDGF–JAK2/STAT3 signaling pathway in RILI and its association with collagen expression. **(A,B)** Western blot analysis of THBS1, PDGFA, JAK2/pJAK2, and STAT3/pSTAT3 protein expression in normal, 20Gy and 30Gy groups of rats; **(C,D)** Western blot analysis of COL6A1, COL6A2, and COL5A1 protein expression in normal and 30Gy groups of rats; **(E)** result of ELISA; **(F)** Pearson correlation analysis (*P < 0.05, **P < 0.01 and ***P < 0.001).

**FIGURE 7:**
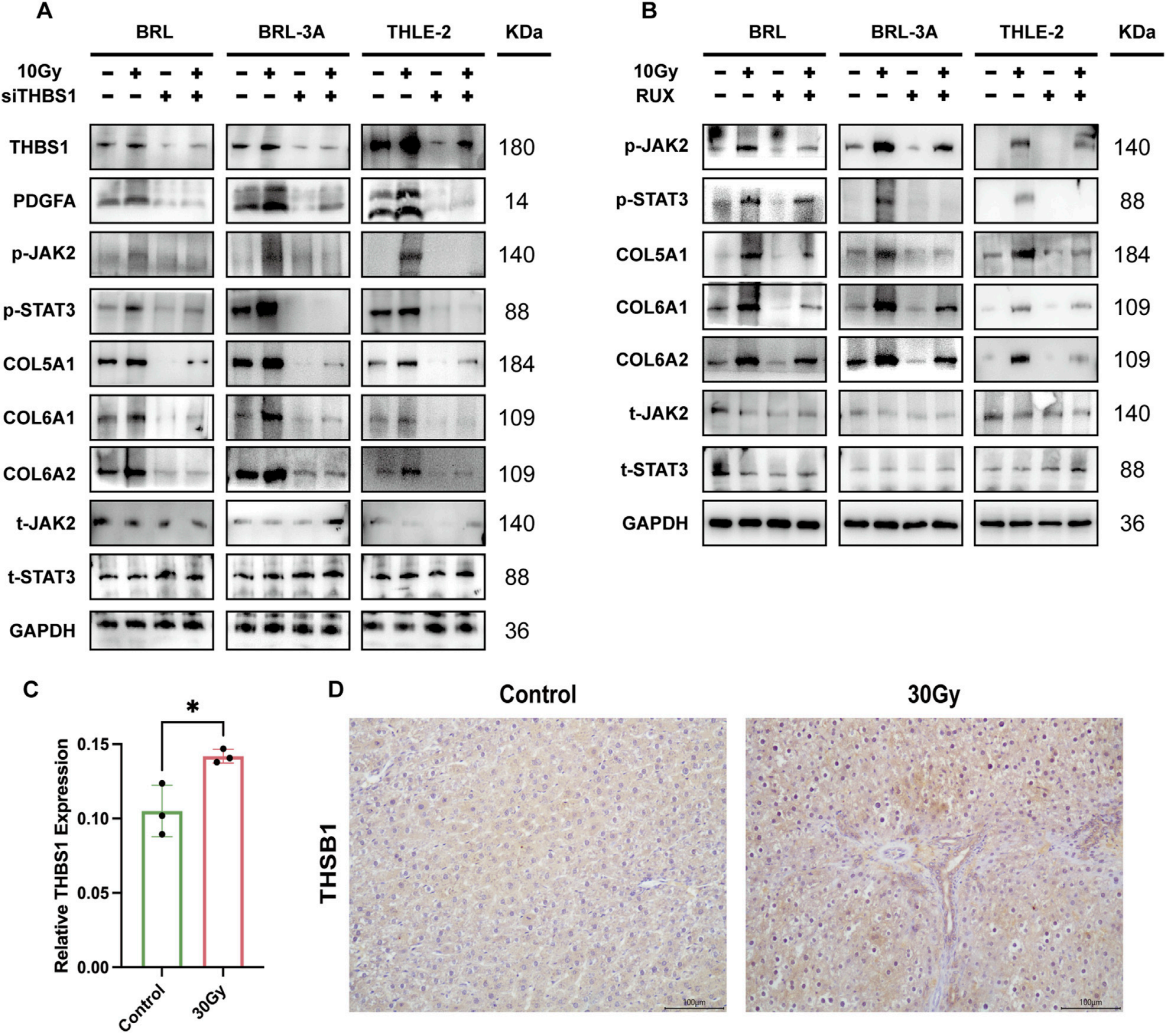
The Effects of siTHBS1 and JAK/STAT Inhibition on JAK2/STAT3 Pathway Activation and Collagen Expression in Radiation-Induced Hepatocytes **(A)** Western blot analysis of THBS1 knockdown in rat (BRL and BRL-3A) and human (THLE-2) hepatocytes shows reduced JAK/STAT activation and reversal of collagen upregulation **(B)** Western blot analysis of JAK/STAT pathway inhibition with Ruxolitinib in hepatocytes, demonstrating reduced JAK2/STAT3 activation and reversal of collagen upregulation; **(C)** Quantitative analysis of THBS1 immunostaining reveals significantly higher expression in the 30 Gy group compared to the controls (P < 0.05); **(D)** Representative IHC images demonstrate increased THBS1 deposition in irradiated (30 Gy) liver tissue compared with controls.

The key protein THBS1, identified through screening, was further validated by WB. As shown in Figures 6A–D, THBS1 expression in the 30 Gy group was significantly higher than that in the control (0 Gy) group and exhibited a clear dose-dependent increase with radiation exposure. The WB results were consistent with the IHC findings (Figures 7A,B), indicating that the radiation-induced upregulation of THBS1 occurred not only at the overall expression level but also within specific tissue regions. IHC analysis further revealed that THBS1 was predominantly localized in ECM of hepatic tissue, particularly within the injured areas, supporting its role as an essential ECM component. Given its potential alteration under pathological conditions, we speculate that THBS1 may serve as a biomarker reflecting disease status. To further assess this hypothesis, THBS1 levels in the plasma of irradiated rats were quantified using an ELISA kit (Animaluni, Shanghai, China). THBS1 concentrations were significantly higher in the 20 Gy and 30 Gy groups than in the control group (P < 0.05; Figure 6E). Moreover, Pearson’s correlation analysis revealed a strong positive association between plasma THBS1 levels and tissue expression (P = 0.0010, R = 0.8973; Figure 6F), indicating that the content of THBS1 assayed in plasma could reflect the degree of RILI in liver tissues to a certain extent.

### 3.8 THBS1 activates the JAK2/STAT3 signaling pathway

WB analysis showed that radiation (30 Gy) significantly increased the expression of PDG-FA, p-JAK2, and p-STAT3 in rat liver tissues, indicating activation of the JAK2/STAT3 pathway (Figures 6A,B). To determine whether this activation was mediated by THBS1, siRNA knockdown of THBS1 in BRL, BRL-3A, and THLE-2 cells markedly reduced p-JAK2 and p-STAT3 levels after irradiation, along with a decline in downstream collagens (COL5A1, COL6A1, COL6A2) (Figure 7). Furthermore, inhibition of JAK2/STAT3 signaling with Ruxolitinib produced similar effects, confi-rming that THBS1 promotes collagen expression through activation of the JAK2/STAT3 pathway.

## 4 Discussion

RILI is a major complication of radiotherapy for HCC and other abdominal or thoracic tumors, often progressing from hepatocyte damage to fibrosis and liver dysfunction (Omiya et al., 2025). T Although oxidative stress and inflammatory cytokine release have been implicated in RILI pathogenesis, the molecular mediators linking radiation exposure to fibrotic remodeling remain unclear (Omiya et al., 2025; Zhou et al., 2024). Our proteomic and network analyses identified THBS1 as a central regulator connecting radiation-induced stress to ECM remodeling. Existing evidence has documents that THBS1 activates profibrotic signaling through TGF-β and PDGF pathways (Murphy-Ullrich, 2019) and our findings extend this understanding by implicating JAK2/STAT3 activation as a downstream mechanism in radiation-induced fibrosis. Moreover, the elevation of plasma THBS1 suggests its potential as a circulating biomarker reflecting the degree of liver injury, providing a new perspective for RILI assessment and therapeutic intervention.

First identified in 1971, THBS1 is a multifunctional matricellular glycoprotein that plays a pivotal role in cell-matrix communication, platelet activation, and vascular homeostasis (Bergseth et al., 2000; Konwar et al., 2025). Under physiological conditions, THBS1 expression in the liver is low, with an average RPKM of 2.825, and its half-life is short due to rapid endothelial degradation (Isenberg and Roberts, 2020). However, radiation exposure markedly enhances its transcription and secretion, indicating that THBS1 is a radiation-responsive ECM regulator rather than a constitutive structural protein. This has been confirmed in our study. Previous studies have shown that THBS1 modulates ECM composition and tissue remodeling by interacting with fibrinogen, fibronectin, and laminin. Excessive THBS1 activity contributes to fibrotic progression in multiple organs (Murphy-Ullrich, 2019; Murphy-Ullrich and Suto, 2018; Petry et al., 1987). IR can alter the epigenome and has long-term effects on gene expression. As evidenced in prior research, THBS1 was in an epigenetically initiated state in fibroblasts with a history of radiation exposure, resulting in sustained upregulation of THBS1 after radiation exposure, impaired fibroblast motility and contractility. Fibroblasts would retain long-term radiation memory in the form of epigenetic alterations. Xu et al. (2025a) demonstrated that THBS1 orchestrates the remodeling of liver organoids by mediating hepatocyte–ECM crosstalk, while hepatocyte-specific THBS1 knockdown improved survival and reduced inflammatory cytokine release in liver failure models (Hassan et al., 2024). Our data extend previous findings. Following THBS1 knockdown via siRNA in the BRL, BRL-3A, and THLE-2 cell lines, we observed suppression of the p-JAK2/p-STAT3 pathway and reduced collagen upregulation. These results suggest that THBS1 acts as both a structural component of the extracellular matrix and a signaling amplifier that links extracellular remodeling to intracellular pro-fibrotic transcription. Along with prior reports on THBS1 promoting multi-organ fibrosis progression, our study confirms the existence of a THBS1-PDGFA-JAK/STAT module that drives radiation-associated matrix deposition. At present, we know little about the role of THBS1, although is widely recognized as a prothrombotic protein. Our study detected significantly elevated THBS1 expression in both tissues and plasma. Tissue expression showed a strong positive correlation with plasma concentrations. Thus, plasma levels can partially reflect tissue THBS1 expression. THBS1 may function as a pivotal hub protein in RILI-induced hepatic fibrosis.

Microvascular damage and ECM remodeling are characteristic changes after radiation exposure. Excessive ECM deposition can lead to the development of fibrosis. As one of the important components of the ECM, collagen is responsible for the formation of the complex extracellular meshwork. Together with COL6A1 and COL6A3, COL6A2 constitutes the triple-helical backbone of collagen VI, a key structural element of the extracellular matrix widely distributed in mammalian tissues. COL6A1 and COL6A2, which are tandemly linked on chromosome 21, are frequently dysregulated under pathological conditions and are markedly upregulated in fibroinflammatory diseases (Rasmussen et al., 2017). COL6 was found to have increased mRNA and protein deposition in the lung of patients with pulmonary fibrosis (Wang et al., 2025), and COL1A1 was highly expressed in RILI (Liu J. et al., 2024). Similarly, our proteomic analysis revealed a significant increase of COL6A2 expression in the rat RILI model, suggesting its potentially key role in the process of RILI.

Radiation exposure markedly increased COL6A1, COL6A2, and COL5A1 expression in rat liver, reflecting early ECM remodeling and the initiation of fibrotic responses. Similar collagen alterations have been reported in diverse fibrotic contexts, where collagen type V and type VI play essential roles in maintaining ECM integrity, regulating fibrillogenesis, and influencing cell–matrix communication. Type V collagen is consistently elevated in liver fibrosis and has been validated as a circulating biomarker reflecting dynamic fibrotic progression (Mak et al., 2016; Vassiliadis et al., 2012). Likewise, collagen VI is increasingly recognized as a stress-responsive ECM molecule that participates in tissue protection and remodeling; its upregulation under ultraviolet irradiation prevents neuronal apoptosis via PI3K/Akt signaling, demonstrating a general role in cytoprotective adaptation to stress (Cheng et al., 2011). In this study,the radiation-associated induction of collagen V and VI likely represents an adaptive remodeling process of the extracellular matrix in response to cellular stress and damage.

Our experiments further demonstrated that silencing THBS1 in both human (THLE-2) and rat (BRL and BRL-3A) hepatocytes reversed the radiation-induced increase of COL6A1, COL6A2, and COL5A1, suggesting that THBS1 promotes ECM gene expression upstream. This finding is consistent with previous research suggesting that THBS1 functions as a potent regulator of tissue repair and fibrosis by facilitating the activation of latent TGF-β and promoting fibroblast activation and collagen synthesis (Petry et al., 1987). Furthermore, inhibiting JAK2/STAT3 signaling with ruxolitinib reversed collagen upregulation, suggesting that THBS1 mediates radiation-induced collagen expression by activating JAK2/STAT3-dependent profibrotic signaling. This interpretation is consistent with the current understanding that radiation-induced fibrosis (RIF) involves sustained cytokine signaling, myofibroblast activation, and ECM accumulation driven by pathways such as PDGF, TGF-β, and JAK/STAT (Kreuder et al., 2025).

Through bioinformatics analysis, DEPs were observed to be mainly enriched in MFs such as heme-binding, ion-binding, and transition metal ion-binding, and THBS1 might act as a key molecule in collagen deposition through heme-binding (Zhang et al., 2022). Binding of THBS1 to calcium ions can change its spatial structure, effectively promoting the binding of THBS1 to cell surface receptors (e.g., β1 integrins and CD47), maintaining the stability of THBS1 (Samsonraj et al., 2023), as well as regulating cell proliferation adhesion migration and tissue repair (Wilson et al., 2024). In the structural domain enrichment analysis, the structural domain of THBS1 was significantly enriched in RILI with a multiplicity of difference Log2FC of 0.6333 (P = 0.04378).

Bioinformatics and network analyses revealed that THBS1 plays a significant role in biological processes and signaling pathways associated with liver injury, suggesting its potential regulatory function in RILI. Rather than acting as an independent effector, THBS1 appears to integrate various injury-related pathways, including platelet degranulation, ECM remodeling, and PDGF signaling. This integration links vascular responses and fibrotic progression. Previous studies have shown that platelet activation and degranulation are among the earliest responses to radiation exposure, leading to the release of growth factors that contribute to inflammation and fibrosis (Asadzadeh-Aghdaei et al., 2019; Makarov et al., 2024).As a platelet-reactive matricellular protein, THBS1 can amplify this response by binding to platelet receptors and promoting further platelet activation (Khan et al., 2023; Gob et al., 2025). The PDGF/PDGFR axis is known to regulate fibroblast proliferation and matrix deposition and has been associated with hepatic and radiation-induced fibrosis (Abderrahmani et al., 2018; Herbst et al., 1995; Partida-Zavala et al., 2018; Kulkarni et al., 2016). Together with prior evidence, our findings suggest that THBS1 may enhance PDGF signaling by facilitating ligand-receptor interaction or stabilizing PDGF activity. This interpretation is consistent with previous reports that THBS1 binds to PDGF and potentiates its downstream profibrotic effects (Belotti et al., 2016). Such interactions could form part of a self-reinforcing loop in which platelet activation, THBS1 secretion, and PDGF signaling collectively promote ECM accumulation and fibrotic remodeling following radiation injury. Therefore, THBS1 could serve as a biomarker to unveil the extent of liver damage and represent a mechanistic link between vascular activation and fibrotic transformation in RILI.

The JAK2/STAT3 pathway is central to radiation-induced inflammatory and fibrotic responses. Previous studies have shown that this signaling cascade is primarily activated by cytokines. Irradiated hepatic non-parenchymal cells release IL-6, TNF-α, and other mediators that increase STAT3 activation. This amplifies inflammatory signaling and drives fibrosis progression. (Zhou et al., 2023; Zhou et al., 2012). Xu et al. observed a significant downregulated STAT3 when cirrhosis was in remission (Xu H. et al., 2025). Blockade of STAT3 signaling pathway could attenuate liver fibrosis (Park et al., 2025), revealing negative correlation between STAT3 activation and the degree of liver injury. Abdel et al. also reported that serum STAT3 concentration and tissue expression were significantly elevated in mice after receiving 7.5 Gy (Abdel-Magied et al., 2025). This study further explores the ECM at the gene level. It reveals that THBS1 is an upstream regulatory hub that responds to radiation stimuli. THBS1 then transduces this signal through the PDGF–JAK2/STAT3 pathway. This ultimately manifests as the transcriptional reprogramming of COL6A1, COL6A2, and COL5A1. This study contributes to the existing literature by identifying THBS1 and the JAK2/STAT3 pathway as promising molecular targets for therapeutic intervention in RILI-associated fibrosis, offering a feasible mechanistic basis for future antifibrotic strategies.

Our study confirms the critical role of THBS1 in RILI and identifies it as a mediator of hepatic fibrosis in RILI through PDGF/PDGFR and JAK2/STAT3 signaling pathways. By protein-protein docking (PPD), this study demonstrated that THBS1 was a key protein in activating the PDGF/PDGFR signaling pathway. Molecular biology verified that THBS1 could mediate hepatic fibrosis by participating in the activation of the PDGF/PDGFR signaling pathway, followed by the activation of the JAK2/STAT3 signaling pathway, ultimately mediating liver fibrogenesis. However, this study still has limitations and constraints. Our findings are based on *in vivo* and *in vitro* experiments, lacking verification from clinical data. Further detection and analysis of the alternations in the expression levels of THBS1 and related signaling pathway in liver tissue and peripheral blood of clinical patients can provide more sufficient evidence for THBS1 as a potential biomarker for RILI. In addition, there are only 3 rats in the experimental group (20Gy, 30Gy) and control group animal models, respectively. Increasing sample size for animal experiments (n ≥ 5/group) can further improve statistical power and result reliability.

Collectively, THBS1 may serve as a potential biomarker for RILI, detected in plasma, for diagnosing and monitoring RILI. Simultaneously, there is a significant positive correlation between THBS1 expression in plasma and tissues, suggesting its clinical value as a potential biomarker for RILI. Nevertheless, owing to the investigation in rat RILI model only, future clinical trials are needed to further validate the diagnostic and therapeutic significance of THBS1 in RILI.

## Data Availability

The data presented in the study are deposited in the ProteomeXchange repository, accession number PXD070440: https://proteomecentral.proteomexchange.org/cgi/GetDataset?ID=PXD070440.
