# Supplementary material for: THBS1 as a candidate biomarker and fibrotic mediator in radiation-induced liver injury: insights from TMT-labeled quantitative proteomics
Source: Front Pharmacol. 2025 Nov 21;16:1659870. doi: 10.3389/fphar.2025.1659870 (PMC12699269; doi:10.3389/fphar.2025.1659870)
Supplement: Supplementary file 1 [file Supplementaryfile1.docx]

Supplementary Material


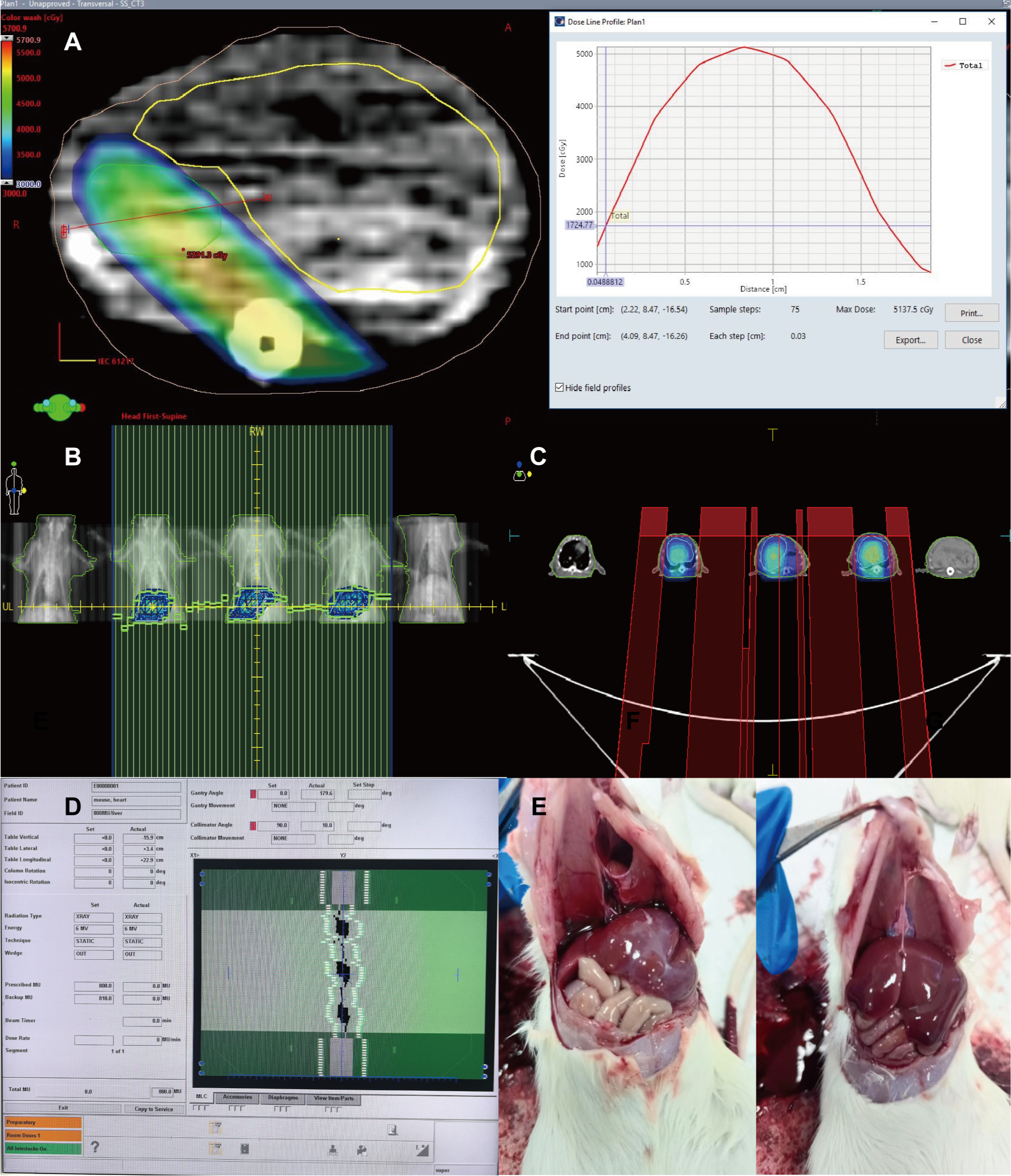


**Supplementary Figure 1:** Rat RILI Modeling. **(A)** The dose distribution map of the 30 Gy irradiation field, where we have drawn a reference line between the liver and adjacent intestinal region, demonstrating the dose drop-off from the liver region to the surrounding intestine. This analysis confirms that the irradiated dose to the intestine is minimal and not significantly affected by the radiation field. **(B)** Depicts the liver irradiation field distribution. **(C)** Illustrates the MLC-shaped irradiation field. **(D)** shows the machine control interface for the irradiation plan executed by the linear accelerator for rat liver irradiation.**(E)**The liver tissue was removed through surgery.


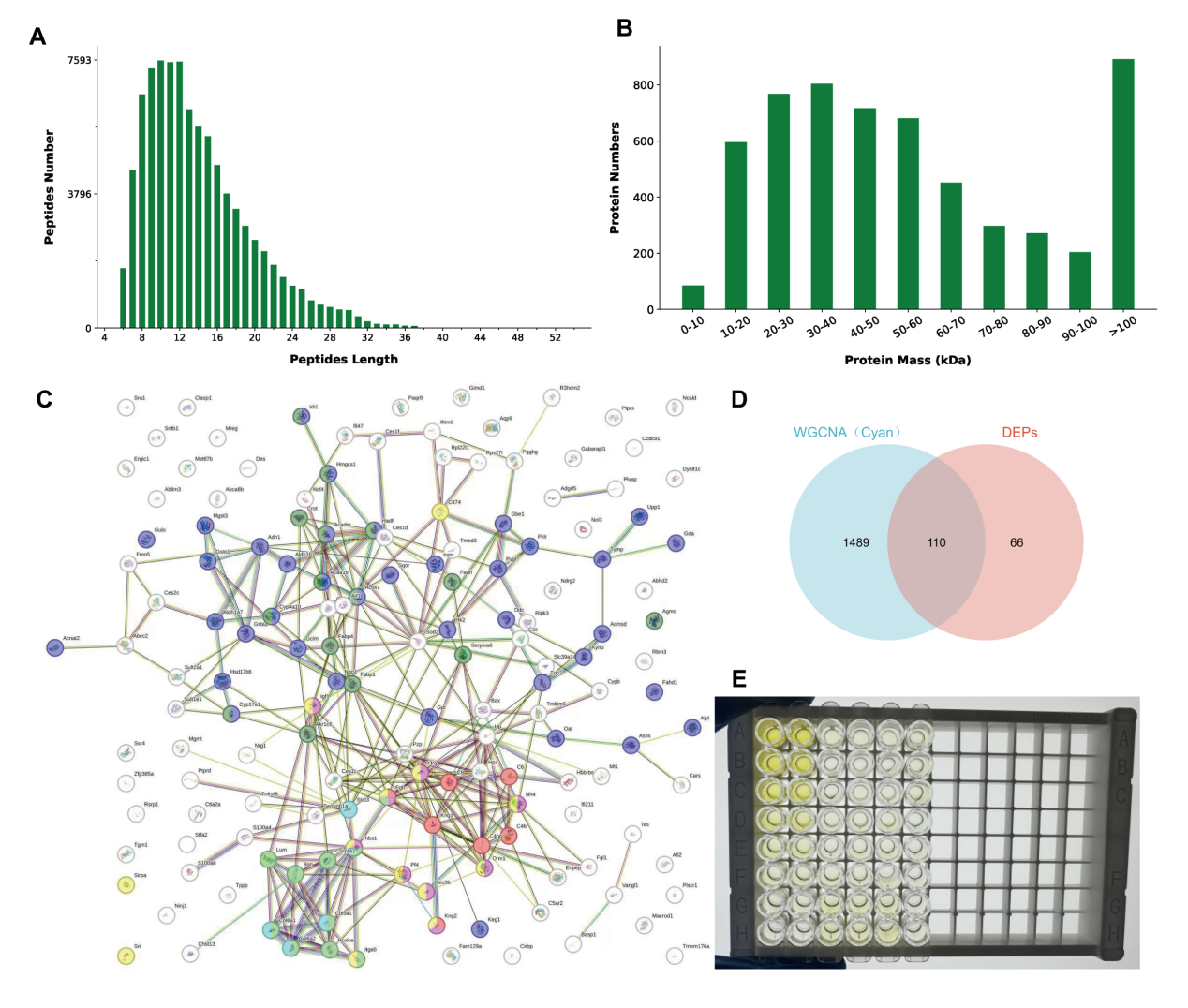


**Supplementary Figure 2:** Characterization of proteomics data. **(A)** Distribution of Precursor Ion Tolerance, reflects high calibration accuracy of the mass spectrometer and supports the overall reliability of peptide identification; **(B)** Distribution of Peptides Number in Different Peptides Length Range; **(C)** PPI analysis of DEGs; **(D)** Venn diagrams for WGCNA and DEPs; E: Results of Plasma Elisa.

**Supplementary Table 2:Antibody information**

| antibody | company | stock number | dilution ratio |
| --- | --- | --- | --- |
| THBS1 | Huabio，China | HA721916 | 1:2000（WB&IHC） |
| PDGFA | Huabio，China | ER1914-91 | 1:500（WB） |
| JAK2 | Huabio，China | ET1607-35 | 1:2000（WB） |
| Phospho-JAK2（Tyr1007、Tyr1008） | Huabio，China | ET1607-34 | 1:1000（WB） |
| STAT3 | Huabio，China | ET1607-38 | 1:2000（WB） |
| Phospho-STAT3（Ser727） | Huabio，China | ET1607-39 | 1:1000（WB） |
| COL5A1 | Proteintech，China | 67604-1-Ig | 1:5000（WB） |
| COL6A1 | Huabio，China | ET1612-91 | 1:2000（WB） |
| COL6A2 | Upingbio，China | YP-mAb-16994 | 1:500（WB） |

**Supplementary Table 2:Drug information**

| Drug | company | stock number | concentration |
| --- | --- | --- | --- |
| Ruxolitinib | Selleckchem Houston, TX, USA | S1378 | 5µM |
